# Isolation and Characterization of Three New Monoterpene Synthases from *Artemisia annua*

**DOI:** 10.3389/fpls.2016.00638

**Published:** 2016-05-10

**Authors:** Ju-Xin Ruan, Jian-Xu Li, Xin Fang, Ling-Jian Wang, Wen-Li Hu, Xiao-Ya Chen, Chang-Qing Yang

**Affiliations:** ^1^National Key Laboratory of Plant Molecular Genetics and National Plant Gene Research Center (Shanghai), CAS Center for Excellence in Molecular Plant Sciences, Institute of Plant Physiology and Ecology, Shanghai Institutes for Biological Sciences, Chinese Academy of Sciences, ShanghaiChina; ^2^Shanghai Key Laboratory of Plant Functional Genomics and Resources, Shanghai Chenshan Plant Science Research Center, Chinese Academy of Sciences, ShanghaiChina

**Keywords:** *Artemisia annua*, monoterpene synthase, β-myrcene, camphene, 1, 8-cineole

## Abstract

*Artemisia annua*, an annual herb used in traditional Chinese medicine, produces a wealth of monoterpenes and sesquiterpenes, including the well-known sesquiterpene lactone artemisinin, an active ingredient in the treatment for malaria. Here we report three new monoterpene synthases of *A. annua*. From a glandular trichome cDNA library, monoterpene synthases of *AaTPS2*, *AaTPS5*, and *AaTPS6*, were isolated and characterized. The recombinant proteins of AaTPS5 and AaTPS6 produced multiple products with camphene and 1,8-cineole as major products, respectively, and AaTPS2 produced a single product, β-myrcene. Although both Mg^2+^ and Mn^2+^ were able to support their catalytic activities, altered product spectrum was observed in the presence of Mn^2+^ for AaTPS2 and AaTPS5. Analysis of extracts of aerial tissues and root of *A. annua* with gas chromatography–mass spectrometry detected more than 20 monoterpenes, of which the three enzymes constituted more than 1/3 of the total. Mechanical wounding induced the expression of all three monoterpene synthase genes, and transcript levels of *AaTPS5* and *AaTPS6* were also elevated after treatments with phytohormones of methyl jasmonate, salicylic acid, and gibberellin, suggesting a role of these monoterpene synthases in plant–environment interactions. The three new monoterpene synthases reported here further our understanding of molecular basis of monoterpene biosynthesis and regulation in plant.

## Introduction

Plants produce a plethora of organic compounds, among which terpenoids constitute the largest group with highly diversified structures and functionality. Apart from a small number of terpenoids that are essential for plant growth and development, the majority functions as specialized (or secondary) metabolites and is involved in the interaction of the plant with its environment (Gershenzon and Dudareva, 2007; Allmann and Baldwin, 2010; Tholl and Lee, 2011), such as phytoalexins against pathogens and herbivores (Ben-Yehoshua et al., 2008; Rodriguez et al., 2011; Schmelz et al., 2011), airborne molecules of plant–plant (Baldwin et al., 2006) or plant–insect signaling (Olson et al., 2008; Allmann and Baldwin, 2010). Terpenoids are not only abundant in many essential oils and resins but also are emitted from the foliage and flower of a variety of plant species (Ahmad and Misra, 1994; Brown et al., 2003; McKay et al., 2003).

*Artemisia annua* is an annual herb of the Asteraceae family. Extensive chemical analyses of plant extracts have demonstrated the presence of several classes of secondary metabolites, including terpenoids and flavonoids (Woerdenbag et al., 1990; Juteau et al., 2002; Lopes-Lutz et al., 2008). Among those with pharmacological activities, the sesquiterpene lactone artemisinin is widely used in the treatment of malaria, especially in the form of combination therapies (van Agtmael et al., 1999; Graham et al., 2010). The volatile blend of *A. annua* comprises both monoterpenes and sesquiterpenes. The monoterpene fraction is composed of a diverse array of structures from the regular and irregular acyclic compounds (e.g., linalool, β-myrcene, and artemisia alcohol) to the monocyclic (e.g., phellandrene and 1,8-cineole), bicyclic (e.g., borneol and camphor), and the tricyclic compounds (e.g., tricyclene; Ahmad and Misra, 1994; Woerdenbag et al., 1994; Brown, 2010).

Plant monoterpenes are usually formed in plastids and their accumulation is often associated with complex secretory or storage structures such as glandular trichomes, secretory cavities, and resin ducts (Byun-McKay et al., 2006; Wang et al., 2008; Goodger et al., 2009; Goodger and Woodrow, 2011; Vitalini et al., 2011). Monoterpenes are derived from the C_10_ precursor of geranyl diphosphate (GPP), catalyzed by monoterpene synthase (Tholl, 2006). Approximately 1/3 of plant monoterpene synthases characterized so far convert GPP into acyclic products (Degenhardt et al., 2009). These reactions proceed by ionization with the assistance of a divalent metal ion (usually Mg^2+^ or Mn^2+^) to the extended geranyl cation, followed by proton loss to form olefinic products including (E)-β-ocimene and β-myrcene or addition of water to form terpene alcohol such as geraniol or linalool. It is also conceivable that linalool, β-myrcene, and (E)-β-ocimene are derived from the linalyl cation that is the result of a previous isomerization. The formation of cyclic products requires reliminary isomerization of the geranyl cation to a linalyl intermediate capable of cyclization to α-terpinyl cation, which is the universal intermediate for the production of cyclic monoterpenes (Bohlmann et al., 1998; Degenhardt et al., 2009).

To date three monoterpene synthases of *A. annua* have been characterized, including two linalool synthases (AaQH1 and AaQH5) and a β-pinene synthase (AaQH6; Jia et al., 1999; Lu et al., 2002). *AaQH1* and *AaQH5* display 88% nucleotide sequence identity with each other and are expressed primarily in leaves and inflorescence but not in root, and the expression is inducible at transcriptional level by mechanical wounding. Although *in vitro* AaQH1 and AaQH5 converted GPP into (*3R*)-linalool, this compound was not detected in the essential oil of *A. annua* leaves (Jia et al., 1999). *AaQH6* showed a circadian pattern of expression and its recombinant protein converted GPP into (-)-β-pinene and (-)-α-pinene at a ratio of 94:6 (Lu et al., 2002). However, most of the monoterpenoids detected in *A. annua* have not been linked to a monoterpene synthase. In this investigation, we cloned and functionally characterized three monoterpene synthases of *A. annua*: AaTPS2, AaTPS5, and AaTPS6, which produce β-myrcene, camphene, and 1,8-cineole as their major products, respectively.

## Materials and Methods

### Plant Materials and Reagents

*Artemisia annua* cv. Qiute was used in this investigation and the seeds were collected from Sichuan Province, China. Seeds of *A. annua* were surface-sterilized and germinated in Murashige and Skoog medium. Seedlings (1 week old) were transferred to soil and grown in greenhouse at 25°C under light intensity of 150 μmol photons m^-2^s^-1^ with 14-h-light/10-h-dark cycle. Tissues from 6-week-old plants were collected for further analysis unless otherwise indicated. To induce flowering, 2-month-old plants were transferred to 12-h-light/12-h-dark photoperiod and inflorescences were collected in the next month. Leaves close to inflorescences (approximately one third of the upper stem) were defined as young leaves, and those close to the basal part (one-third of the lower stem) were defined as mature leaves. All the biochemicals and reagents were purchased from Sigma–Aldrich (St. Louis, MO, USA), unless otherwise noted.

### Phytohormone and Wounding Treatment

For salicylic acid (SA), methyl jasmonate (MeJA), and gibberellin (GA) treatments, 4-week-old plants of *A. annua* were dipped in the phytohormone solution (5 mM of SA, 50 μM of MeJA, or 100 μM of GA) or dimethyl sulfoxide (DMSO) solution for 4 h. Mechanical wounding of 4-week-old plants was conducted as published (Lewinsohn et al., 1992). Young leaves were collected and total RNAs were isolated from three treated individual plants for analysis.

### Plant Terpenoids Extraction

Fresh plant materials (0.5 g) were collected and ground with liquid nitrogen and extracted with 2.5 ml pentane containing 2 ng/μl nonyl acetate in a shaker at 28°C for 1 h. The extractions were analyzed by gas chromatography–mass spectrometry (GC–MS; Agilent 6890 Series GC System coupled to an Agilent 5973 Network Mass Selective Detector), with the temperature program: initial temperature of 40°C (5 min hold), increase to 160°C at 10°C/min, and ramp to 280°C at 30°C/min (5 min hold). Products were identified by comparison with authentic standards and NIST (National Institute of Standards and Technology) and Wiley libraries.

### Gene Isolation, Expression, and Sequence Analysis

Total RNA was extracted using TRIzol^®^ reagent (Thermo Scientific, Waltham, MA, USA) and 1 μg total RNA was reverse-transcribed using the RNA PCR kit (TaKaRa, Dalian, China), followed by cDNA synthesis and gene expression analysis. Full length cDNAs were isolated by 5′- and 3′-rapid amplification of cDNA ends (5′-and 3′- RACE) using the Pfu DNA polymerase (Promega, Fitchburg, WI, USA). Quantitative real-time PCR (qRT-PCR) was performed with SYBRGreen PCR Mastermix (TaKaRa, Dalian, China) on a Mastercycler^®^ epRealPlex2 (Eppendorf, Hamburg, Germany) cycler with *A. annua actin* (EU531837) as reference. Transcript levels of genes were determined as described previously (Yu et al., 2010). Nucleotide and amino acid sequence alignments were performed using ClustalW^1^. Chloroplast signal peptide prediction was performed at ChloroP^2^ and SignalP^3^ websites. Primers used in this investigation are listed in Supplementary Table 1.

### Prokaryotic Expression and Protein Purification

To facilitate prokaryotic expression, the N-terminal signal peptide of AaTPS2, AaTPS5, and AaTPS6 (46, 57, and 46 amino acid residues, respectively) before RR motif (Lin et al., 2008) was truncated by PCR amplification with Pfu DNA polymerase (Supplementary Table 1). PCR products were digested by *Nco*I and *Sal*I and ligated into pET-32a expression vector (Novagen, Darmstadt, Germany). The resulting plasmids were confirmed by sequencing and were transferred into *Escherichia coli* BL21 (DE3). *E. coli* cells harboring expression vectors were grown at 37°C till OD_600_ = 0.5, and protein production was induced by 1 mM isopropyl beta-D-1-thiogalactopyranoside (IPTG) at 22°C for 24 h. Recombinant proteins were purified with Ni-NTA resin according to manufacturer’s manual (Qiagen, Hilden, Germany). The protein concentration was determined using the Bradford method (Bradford, 1976).

### Enzyme Assay

Assays of catalytic activities of recombinant proteins were performed in a volume of 500 μl reaction buffer (25 mM HEPES, pH 7.0, 5 mM MgCl_2_, 5 mM dithiothreitol), containing 40 μM GPP and 10 μg protein, at 30°C for 1 h unless otherwise indicated. The reaction mixture was extracted with 500 μl pentane and subjected to analysis by GC–MS as described above. For quantitative analysis, nonyl acetate was added as internal standard during pentane extraction of the enzyme reaction mixture. The kinetic parameters of recombinant AaTPS2, AaTPS5, and AaTPS6 were determined according to previously published (Kampranis et al., 2007). Briefly, 3 μg of the purified enzyme was added to each assay mixture containing GPP ranging from 3 to 100 μM, and incubated at 30°C for 5 min, then stopped by the addition of 0.5 M EDTA (pH 8.0). The reaction products were extracted with 500 μl pentane containing 2 ng/μl nonyl acetate, followed by GC–MS analysis. Kinetic parameter values were obtained with GraphPad Prism 5 software (GraphPad Software, Inc.). To determine the optimal temperature, the assays were conducted at a series of temperatures ranging from 25 to 45°C.

## Results

### Monoterpenes and Sesquiterpenes in *A. annua* Tissues

**FIGURE 1:**
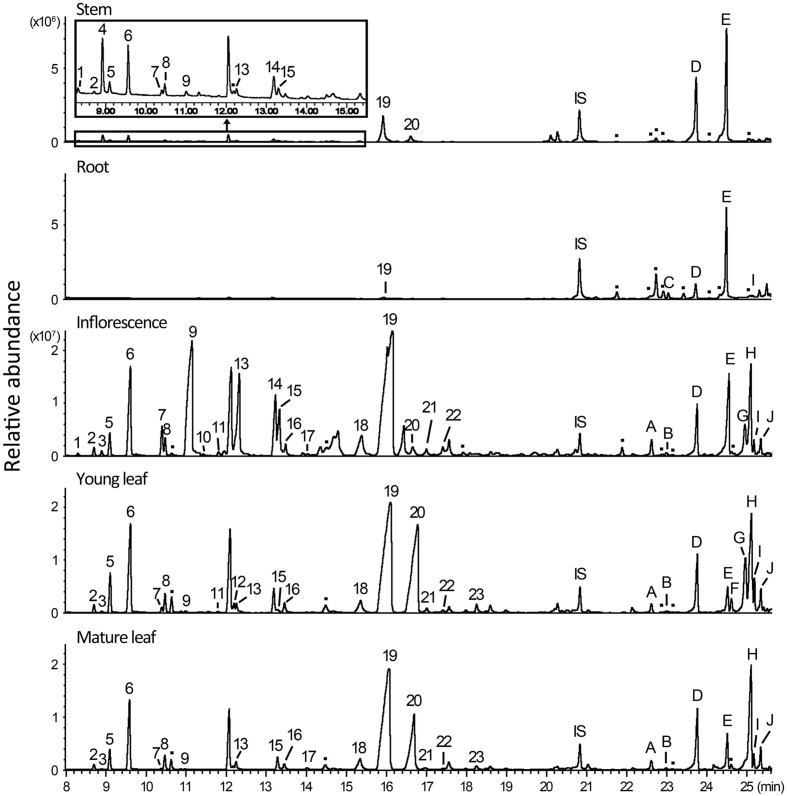
**Monoterpenes and sesquiterpenes produced by *Artemisia annua* plant.** Fresh samples of stem, root, inflorescence, young leaf, and mature leaf were extracted with *N*-pentane containing 2 ng/μl nonyl acetate in a shaker at 28°C for 1 h and analyzed by gas chromatography–mass spectrometry (GC–MS). Peaks are: IS: Internal Standard, nonyl acetate; peaks 1–23 are monoterpenes: (1) santolina triene; (2) tricyclene; (3) α-thujene; (4) artemisia triene; (5) α-pinene; (6) camphene; (7) sabinene; (8) β-pinene; (9) β-myrcene; (10) I-phellandrene; (11) α-terpinene; (12) limonene; (13) 1,8-cineole; (14) γ-terpinene; (15) artemisia ketone; (16) *trans*-sabinene hydrate; (17) artemisia alcohol; (18) chrysanthenone; (19) camphor; (20) borneol; (21) 4-terpineol; (22) α-terpineol; (23) *trans*-carveol; peaks A–H are sesquiterpenes: (A) α-copaene; (B) β-cubebene; (C) β-elemene; (D) *trans*-caryophyllene; (E) *trans*-β-farnesene; (F) γ-curcumene; (G) naphthalene; (H) germacrene D; (I) β-selinene; (J) bicyclogermacrene; peaks labeled with dots represent monoterpenes and sesquiterpenes that were not unambiguously identified.

To analyze monoterpenes and sesquiterpenes in different parts of *A. annua*, fresh tissues of root, young and mature leaf, stem, and inflorescence were extracted with *N*-pentane and subjected to GC–MS. Totally, there were 23 monoterpenes and 10 sesquiterpenes being detected and identified by comparison with authentic standards and GC–MS database, and, additionally, there were at least three monoterpenes and eight sesquiterpenes that were detectable from the extracts but could not be identified unambiguously (**Figure 1**).

Of the five tissues examined, inflorescence contained the most abundant and diverse terpenes. In total, 23 monoterpenes (including three unidentified) and 12 sesquiterpenes (including four unidentified) were detected in inflorescence extracts, amounting up to 4 mg/g fresh weight (FW; **Figure 1** and Supplementary Table 2). The total contents of monoterpenes varied significantly in different organs (i.e., >100-fold higher in inflorescence than in root), in comparison to the less degree of variations of total sesquiterpene contents in these organs (Supplementary Table 2). Among the monoterpenes calculated, only camphor was detected in all tissues examined, and was also the only monoterpene detected in root (**Figure 1**). Others like artemisia triene, β-myrcene, and *trans*-carveol, showed distinct distribution patterns. For example, artemisia triene was most abundant in stem; and β-myrcene was the major component in inflorescence but a minor one in leaf, whereas *trans*-carveol was detected only in leaf. Generally, inflorescence has more abundant monoterpenes but shares a similar spectrum with leaf (**Figure 1**). In comparison with monoterpenes, sesquiterpenes were more diversified, among which β-farnesene was abundant in root and stem, and germacrene D accumulated mainly in leaf and inflorescence (**Figure 1**).

### Isolation of Monoterpene Synthase Genes

A cDNA library of *A. annua* glandular trichome (Li et al., 2013) was searched for monoterpene synthase genes based on both annotation and sequence comparisons, and full-length cDNAs were obtained by 5′- and 3′-rapid amplification of cDNA ends (RACE). Three cDNAs, namely *AaTPS2* (KF987082), *AaTPS5* (KF987083), and *AaTPS6* (KF987084), encoding proteins of 586, 602, and 587 amino acids, respectively, were isolated. Searching of NCBI non-redundant protein database revealed that these proteins share the highest sequence identities with plant monoterpene synthases, including AaQH1, AaQH5, and AaQH6 previously reported (Jia et al., 1999; Lu et al., 2002). AaTPS5 has a protein sequence identity of 54% with AaQH6, whereas AaTPS2 and AaTPS6 are over 65% identical to AaQH1 and AaQH5 (Jia et al., 1999; Lu et al., 2002).

**FIGURE 2:**
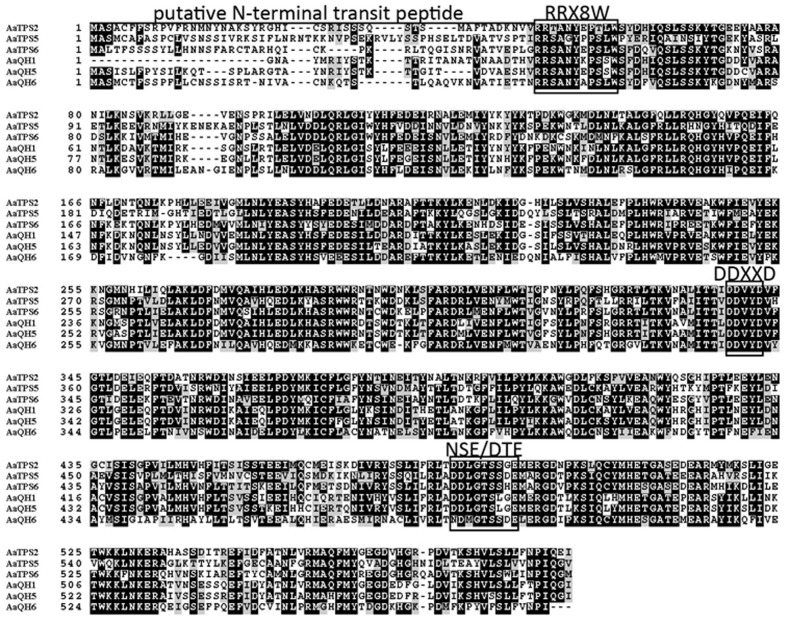
**Alignment of deduced amino acid sequences of AaTPS2, AaTPS5, and AaTPS6 with linalool synthases (AaQH1 and AaQH5) and β-Pinene synthase (AaQH6) of *A. annua*.** The horizontal line marks the putative *N*-terminal transit peptide. The conserved RRX8W and DDXXD motifs and the additional metal binding NSE/DTE domain are marked with frames. The alignment was carried out by Clustal Omega (http://www.ebi.ac.uk/Tools/msa/clustalo/) and the result of alignment was formatted by BoxShade (http://www.ch.embnet.org/software/BOX_form.html).

Alignment of these new monoterpene synthases with AaQH1, AaQH5, and AaQH6 showed that, besides the putative plastid targeting signaling sequence at N-terminal, the DDxxD domain involved in metal cofactor binding are present in all six proteins (**Figure 2**). There is also an additional metal binding motif NSE/DTE domain, as well as an RRx_8_W domain that is conserved in plant monoterpene synthases (**Figure 2**). Phylogenetic analysis of AaTPS2, AaTPS5, and AaTPS6 with terpene synthases from other plant species placed the three in the TPS-b subfamily, along with other angiosperm monoterpene synthases (Supplementary Figure 1; Bohlmann et al., 1998).

### Enzymatic Activities of AaTPS2, AaTPS5, and AaTPS6

**FIGURE 3:**
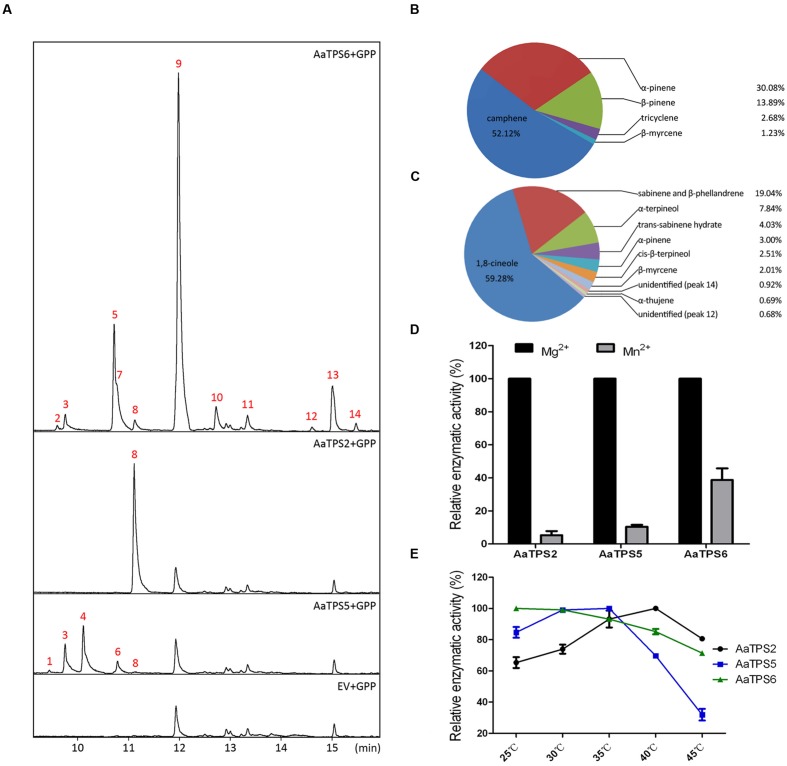
**Enzymatic characterization of AaTPS2, AaTPS5, and AaTPS6 recombinant proteins. (A)** GC–MS analysis of pentane extracts of AaTPS6, AaTPS2, and AaTPS5 recombinant proteins after incubation with GPP as substrate. The protein tag produced by pET32a empty vector was used as control. **(B,C)** Product percentages of AaTPS5 **(B)** and AaTPS6 **(C)**, sabinene (peaks 5) and β-phellandrene (peak 7) were calculated together because they could not be separated well by gas chromatography. Peaks are: (1) tricyclene; (2) α-thujene; (3) α-pinene; (4) camphene; (5) sabinene; (6) β-pinene; (7) β-phellandrene; (8) β-myrcene; (9) 1,8-cineole; (10) *trans*-sabinene hydrate; (11) *cis*-β-terpineol; (13) α-terpineol; (12) and (14) products unidentified. **(D)** Relative activities of AaTPS2, AaTPS5, and AaTPS6 with different divalent metal cofactors. **(E)** Relative activities of AaTPS2, AaTPS5, and AaTPS6 recombinant proteins toward GPP substrate at different temperatures.

To elucidate their functions, *AaTPS2*, *AaTPS5*, and *AaTPS6* were expressed in *E. coli* after removing the N-terminal plastid targeting sequences. The fusion proteins were purified and incubated with GPP, farnesyl diphosphate (FPP), or geranylgeranyl diphosphate (GGPP), respectively, with Mg^2+^ as metal cofactor. With GPP substrate, AaTPS2 catalyzed the formation of a single monoterpene product, β-myrcene, whereas multiple products were identified for both recombinant AaTPS5 and AaTPS6 (**Figure 3A**). AaTPS5 catalyzed the production of five monoterpenes, of which camphene was the major one which accounted for 52.12% of the total, and (-)-α-pinene (30.08%), (-)-β-pinene (3.89%), tricyclene (2.68%), and β-myrcene (1.23%) were the less abundant products (**Figure 3B**). AaTPS6 also formed multiple products with 1,8-cineole as the major one (59.28% of the total), in addition to ten other products including sabinene and β-phellandrene (together 19.04%), α-terpineol (7.84%), *trans*-sabinene hydrate (4.03%), (-)-α-pinene (3.00%), *cis*-β-terpineol (2.51%), β-myrcene (2.01%), α-thujene (0.69%), as well as two unidentified minor products (**Figure 3C**).

Besides GPP, the recombinant AaTPS5 also converted FPP to β-caryophyllene, but with low catalytic activity (Supplementary Figure 2), whereas neither AaTPS2 nor AaTPS6 showed any detectable activity toward FPP. None of these enzymes were able to accept GGPP as substrate in our assay conditions.

Most of the reaction products isolated from *in vitro* enzymatic assays were detected in *A. annua* extracts except β-phellandrene, *cis*-β-terpineol and two unidentified minor products of AaTPS6. Among the *in vitro* products of the three monoterpene synthases, some such as (-)-α-pinene and β-myrcene, were shared by AaTPS5 and AaTPS6, whereas others like (-)-β-pinene and camphene were common to AaQH6 or AaTPS2. Thus these enzymes have overlapping activities in terms of products, although their *in planta* products could differ. However, the ratio of camphene to tricyclene produced by AaTPS5 (~19:1) was similar to that detected in all plant tissues (17~18:1) except stem (14:1). Similarly, in the main products of AaTPS6 1,8-cineole and sabinene showed a ratio of about 3:1, close to 2.8~3.2:1 in extracts of young leaf and stem, but different form that in inflorescence (~4.8:1; Supplementary Table 3 and **Figure 3A**).

In the absence of a divalent metal ion, recombinant proteins of these three monoterpene synthases showed no activity during incubation with GPP, and the activities were restored when either Mg^2+^ or Mn^2+^ was added as the metal cofactor. All three enzymes exhibited higher catalytic activities with Mg^2+^ than with Mn^2+^ at 5 mM (**Figure 3D**). Unexpectedly, in the presence of Mn^2+^ as the divalent ion, AaTPS2 and AaTPS5 catalyzed the formation of linalool from GPP (Supplementary Figure 3), which was not present in the products extracted from the Mg^2+^-containing reaction buffer (**Figure 3A**).

Kinetic analysis with GPP in the presence of Mg^2+^ showed that AaTPS2 had a Michaelis constant (*K*_m_) of 8.25 μM with estimated *k*_cat_ of 0.52 s^-1^ and a specific constant (*k*_cat_/*K*_m_) value of 6.31 × 10^4^ s^-1^ •M^-1^. The *K*_m_ values of AaTPS5 and AaTPS6 were 19.47 μM and 17.70 μM with estimated *k*_cat_ of 1.49 s^-1^ and 4.46 s^-1^, and *k*_cat_/*K*_m_ values of 7.66 × 10^4^ s^-1^ •M^-1^ and 2.52 × 10^5^ s^-1^ •M^-1^, respectively, (**Table 1**). The *K*_m_ values of AaTPS2, AaTPS5, and AaTPS6 are in the typical range of enzymes involved in plant secondary metabolism (Bar-Even et al., 2011).

**Table 1:** Kinetic parameters of AaTPS2, AaTPS5, and AaTPS6 proteins.

	*K*_m_ (μM)	*K*_cat_ (s^-1^)	*K*_cat_/*K*_m_ (s^-1^ •M^-1^)
AaTPS2	8.25	0.52	6.31 × 10^4^
AaTPS5	19.74	1.49	7.66 × 10^4^
AaTPS6	17.70	4.46	2.52 × 10^5^

The optimum temperatures were determined for the recombinant enzymes at the range from 25 to 45°C. The enzymatic activity of AaTPS6 did not differ significantly in this temperature range: it reached peak at 25°C, and retained 70% at 45°C (**Figure 3E**). The optimum temperatures of AaTPS5 and AaTPS6 were 35 and 40°C, respectively, and decreased rapidly with the rising temperature (**Figure 3E**).

### Expression Patterns of *AaTPS2*, *AaTPS5*, and *AaTPS6*

**FIGURE 4:**
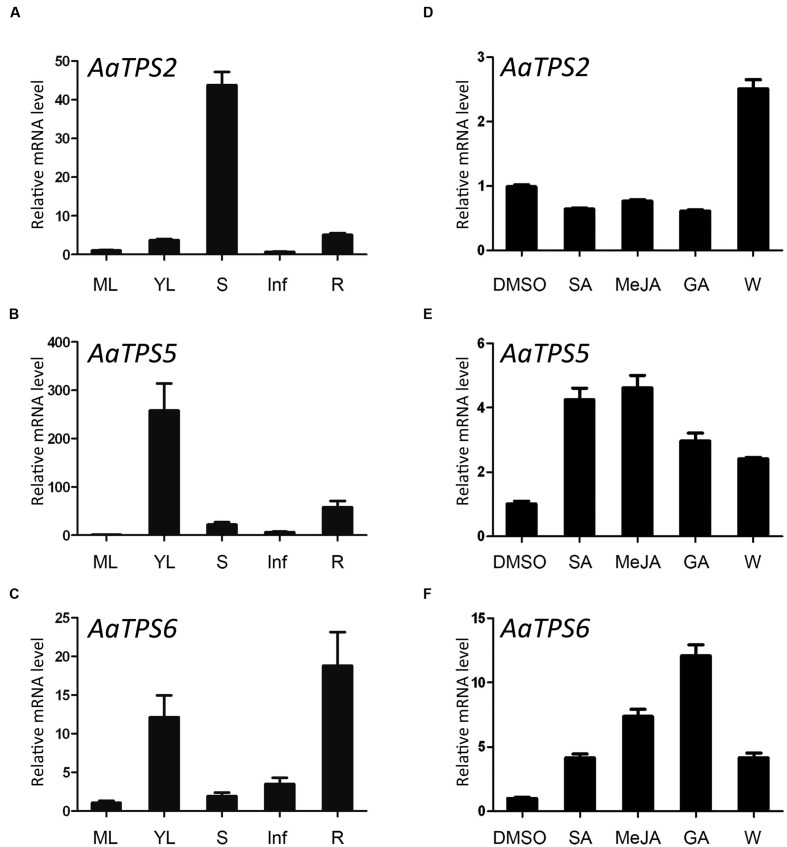
**Expression patterns of *AaTPS2*, *AaTPS5*, and *AaTPS6*.** The transcripts were analyzed by quantitative real-time RT-PCR, with *AaACTIN* (EU531837) as internal standard. **(A–C)**, relative transcript levels of *AaTPS2*, *AaTPS5*, and *AaTPS6* in different organs. ML, mature leaves; YL, young leaves; S, stem; Inf, inflorescence; R, root. **(D–F)** Relative transcript levels of *AaTPS2*, *AaTPS5*, and *AaTPS6* after phytohormone treatments [5 mM salicylic acid (SA), 50 μM methyl jasmonate (MeJA), or 100 μM gibberellin (GA)] and mechanical wounding treatment (W) for 4 h. Error bars indicate standard deviation (SD) of three biological replicates.

Expressions of *AaTPS2*, *AaTPS5*, and *AaTPS6* in leaf, stem, root, and inflorescence were analyzed by qRT-PCR. Although transcripts of these monoterpene synthase genes were detected in all these organs, their expression patterns differed. *AaTPS2* exhibited the highest expression level in stem and low in other tissues; transcript of *AaTPS5* was more abundant in young leaves than in mature leaves; and *AaTPS6* was highly expressed in root and young leaves (**Figures 4A–C**). Despite their divergence in relative transcript levels in different tissues, all these monoterpene synthase genes showed higher expression levels in young leaf than in mature leaf, consistent with the contents of monoterpenes (**Figure 1**).

*AaADS*, encoding a sesquiterpene synthase for the synthesis of amorpha-4,11-diene, a key precursor of artemisinin biosynthesis, shows an increase of steady-state mRNA level upon treatments with phytohormones of SA, MeJA, and GA (Yu et al., 2012). Interestingly, treatment of these phytohormones also induced expressions of *AaTPS5* and *AaTPS6*, but did not affect *AaTPS2* expression (**Figures 4D–F**). SA and MeJA showed similar effects on *AaTPS5* expression (~fourfold increases of transcript level) whereas GA was to a less extent (~threefold increase; **Figure 4E**). *AaTPS6* was induced strongly by GA (~12-fold increase), moderate by MeJA (~eightfold) and less by SA (~fourfold; **Figure 4F**). Moreover, *AaTPS2*, *AaTPS5*, and *AaTPS6* were all up-regulated after mechanical wounding (**Figures 4D–F**), similar to the two linalool synthase genes *AaQH1* and *AaQH5* (Jia et al., 1999; Lu et al., 2002). These different responses of *AaTPSs* to phytohormone and wounding treatments indicate distinct roles of their monoterpene products in *A. annua* plant.

## Discussion

Monoterpenes represented more than 65% of the leaf volatiles and 80% of the inflorescence volatiles as quantified here (Supplementary Table 2). Analyzing of the volatile bouquets of *A. annua* demonstrated that monoterpenes including 1,8-cineole, β-myrcene, (-)-α-pinene, (-)-β-pinene, sabinene, and camphene are the main compounds that contribute to the fragrant odor (Ahmad and Misra, 1994). The monoterpene synthases, AaTPS2, AaTPS5, and AaTPS6 we characterized here are responsible for more than 1/3 monoterpenes produced *in planta*, most of which are bioactive compounds both *in vivo* and *in vitro*. For example, 1,8-cineole, the main product of AaTPS6, is characterized as a mosquito feeding and ovipositional repellent in tarweed (*Hemizonia fitchii*), and also has an effect on progeny production of *Tribolium castaneum* (Klocke et al., 1987; Tripathi et al., 2001). Thus the three monoterpene synthases reported here not only enrich our knowledge of terpene biosynthesis, but also provide gene resources for engineering of bioactive monoterpenes.

Most monoterpenes produced by AaTPS2, AaTPS5, and AaTPS6 are detected in inflorescence and leaf, with comparable proportions of these compounds *in vivo* and *in vitro*. However, discrepancy was found between the transcript levels of these genes and the accumulation of corresponding monoterpenes. In root, the only monoterpene detected was camphor, but *AaTPS2*, *AaTPS5*, and *AaTPS6* were all actively transcribed. It will be interesting to examine if these monoterpenes were indeed produced in root but subjected to secondary modifications, such as oxidation and glycosylation. As previously reported, the yield of terpenoids in aerial organs in various plants is highly dependent on trichome abundance (Biswas et al., 2009). Glandular trichomes of *A. annua* are extensively distributed on aerial organs and their density are higher in young than in older leaves (Olofsson et al., 2011), which is consistent with monoterpene contents detected here.

Among the three monoterpene synthases elucidated here, the recombinant AaTPS2 catalyzes GPP to acyclic β-myrcene as its only product while AaTPS5 and AaTPS6 produce multiple cyclic products with the acyclic β-myrcene as a byproduct. Acyclic monoterpenes, such as β-myrcene and (E)-β-ocimene, may arise by deprotonation of carbocations, whereas the isomerization step to linalyl diphosphate is required in the case of cyclic types, such as limonene and pinenes, which cannot be derived directly from GPP because of the geometric impediment of the *trans*-double bond at C2-C3 (Croteau et al., 1985, 1987). Thus, the differences between the mechanisms in formation cyclic and acyclic monoterpenes are correlated to the production of single or multiple products by different monoterpene synthases, which are capable of overcoming the topological impediment to direct cyclization of GPP initiated by divalent metal ion-dependent ionization (Schwab et al., 2001). Terpene synthases require divalent metal ions as cofactor that binds to the active site during catalysis and different divalent ion metals and concentrations can affect enzyme activities *in vitro* (Picaud et al., 2005). Of the two divalent ion metal tested here, Mg^2+^ is preferred to Mn^2+^ by all three enzymes. Notably, AaTPS2 and AaTPS5 produced an additional acyclic product, linalool, in the presence of Mn^2+^, which accounted for the major product of the corresponding reactions. Although we cannot confidently elucidate the mechanism of reaction process affected by either metal ion, the phenomenon might be due to the alteration of catalytic pocket by binding of lager Mn^2+^ to allow a water molecule to enter the pocket and specifically attack linalyl cation resulted in linalool production. Additional modeling and mutagenesis work shall help to understand the structural basis for these divalent cation dependent catalytic differences.

Many terpenes are important compounds involved in plant tolerance/resistance to biotic and abiotic stresses (Kang et al., 2010; Rodriguez et al., 2011). The mechanical wounding can lead to enhanced expression of monoterpene synthase genes of *A. annua*, suggesting that they are likely involved in an inducible defense system. *AaTPS5* and *AaTPS6* can also be induced by phytohormones including MeJA, SA, and GA, which are important regulators of plant defense against herbivores and pathogens, and modulate epidermal differentiation programs (Jiang and Fu, 2007; Tsuda et al., 2008; Gao et al., 2011; Hou et al., 2013; Yi et al., 2014). The significant induction of *AaTPS6* by GA is consistent with the study of *Salvia officinalis* that 1,8-cineole synthase and its products are induced upon GA treatment (Schmiderer et al., 2010). Our results of the induction pattern of monoterpene synthase genes in *A. annua* provide further clues to the physiological functions of terpenes on plant adaptation.

Although *AaTPS2*, *AaTPS5*, and *AaTPS6* in *A. annua* are responsible for most of the fragrant odor monoterpenes in this medicinal herb, there are still monoterpenes in *A. annua* that have not be ascribed to any of the monoterpene synthases reported so far. This terpenoids-rich herb must have additional monoterpene synthases that await characterization.

## Author Contributions

All the authors conceived and designed the experiments. J-XR, J-XL, XF, L-JW, and W-LH performed the experiments and analyzed the data. J-XR, X-YC, and C-QY wrote the manuscript. All authors approved the final version of manuscript to be published.

## Conflict of Interest Statement

The authors declare that the research was conducted in the absence of any commercial or financial relationships that could be construed as a potential conflict of interest.

## Footnotes

1 http://www.genome.jp/tools/clustalw/

2 http://www.cbs.dtu.dk/services/ChloroP/

3 http://www.cbs.dtu.dk/services/SignalP/
