# Supplementary material for: Isolation and Characterization of Three New Monoterpene Synthases from Artemisia annua
Source: Front Plant Sci. 2016 May 10;7:638. doi: 10.3389/fpls.2016.00638 (PMC4861830; doi:10.3389/fpls.2016.00638)
Supplement: Supplementary file 1 [file Image_1.PDF]

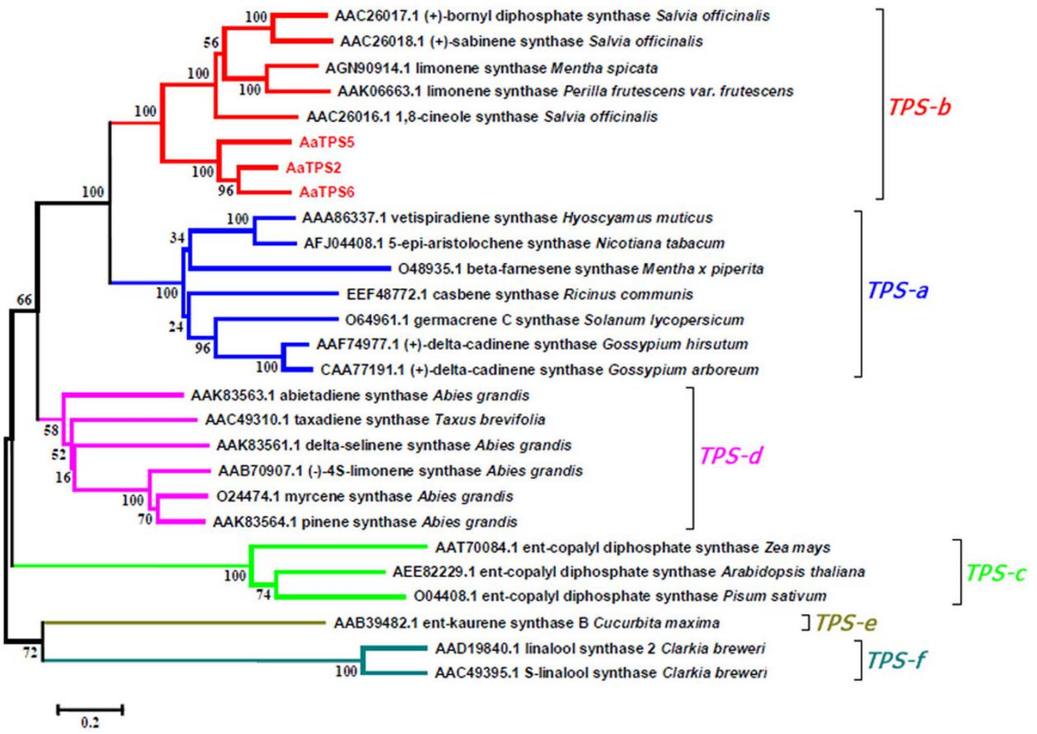


**Supplementary Figure 1. Phylogenetic analysis of AaTPS2, AaTPS5 and AaTPS6 with other terpene synthases.**

Phylogenetic tree was constructed according to Dayhoff's distances between proteins using the neighbor-joining method. Scale bar indicates 0.2 amino acid substitutions per site. Numbers are bootstrap values of each branch.

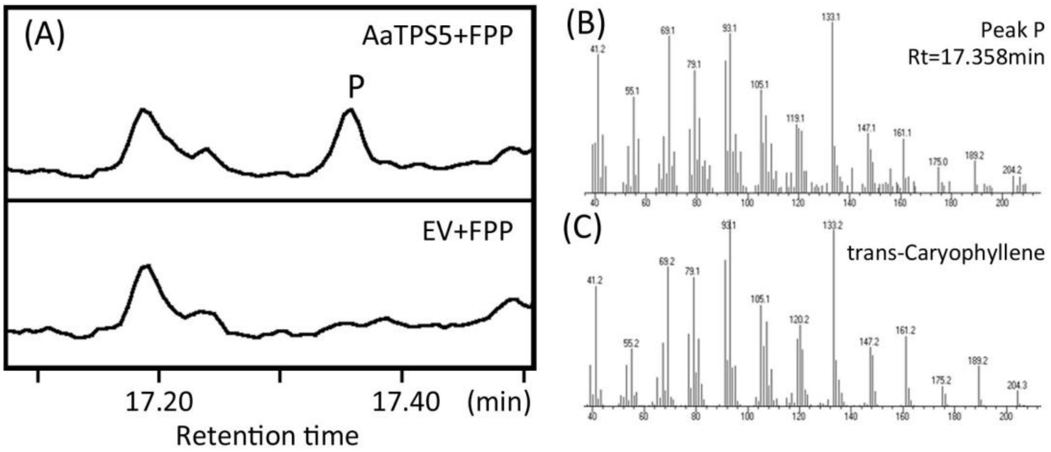


**Supplementary Figure 2. Mass spectra of product formed by recombinant AaTPS5 protein with FPP as substrate.**

(A) GC separation of the product by AaTPS5 protein.

(B) Mass spectrum of AaTPS5 product.

(C) Mass spectrum of authentic standard of trans-caryophyllene.

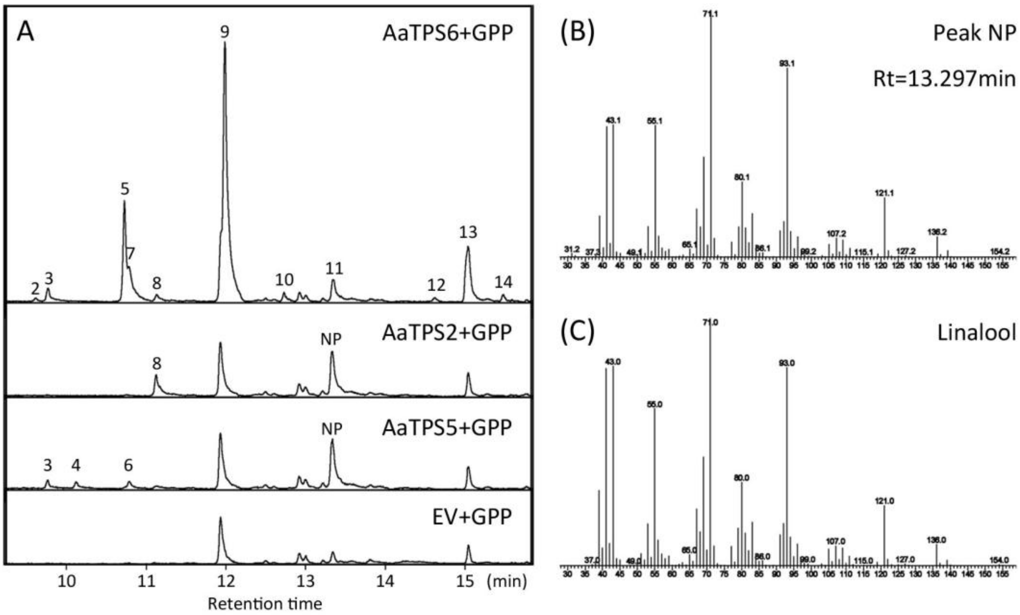


**Supplementary Figure 3. Products of AaTPS2, AaTPS5 and AaTPS6 with 5 mM $Mn^{2+}$.**

(A) GC separation of products of AaTPS6, AaTPS2, AaTPS5. Peaks are: 2, α-thujene; 3, α-pinene; 4, camphene; 5, sabinene; 6, β-pinene; 7, β-phellandrene; 8, β-myrcene; 9, 1,8-cineole; 10, trans-sabinene hydrate; 11, cis- β-terpineol; 13, α-terpinoid; 12 and 14, products unidentified; NP, new product produced by AaTPS2 and AaTPS5.

(B) Mass spectrum of additional product (NP) of AaTPS2 and AaTPS5 with $Mn^{2+}$ as cofactor.

(C) Mass spectrum of authentic standard of linalool.
