# Supplementary figures and images for: Isolation and Characterization of Three New Monoterpene Synthases from Artemisia annua
Source: Front Plant Sci. 2016 May 10;7:638. doi: 10.3389/fpls.2016.00638 (PMC4861830; doi:10.3389/fpls.2016.00638)

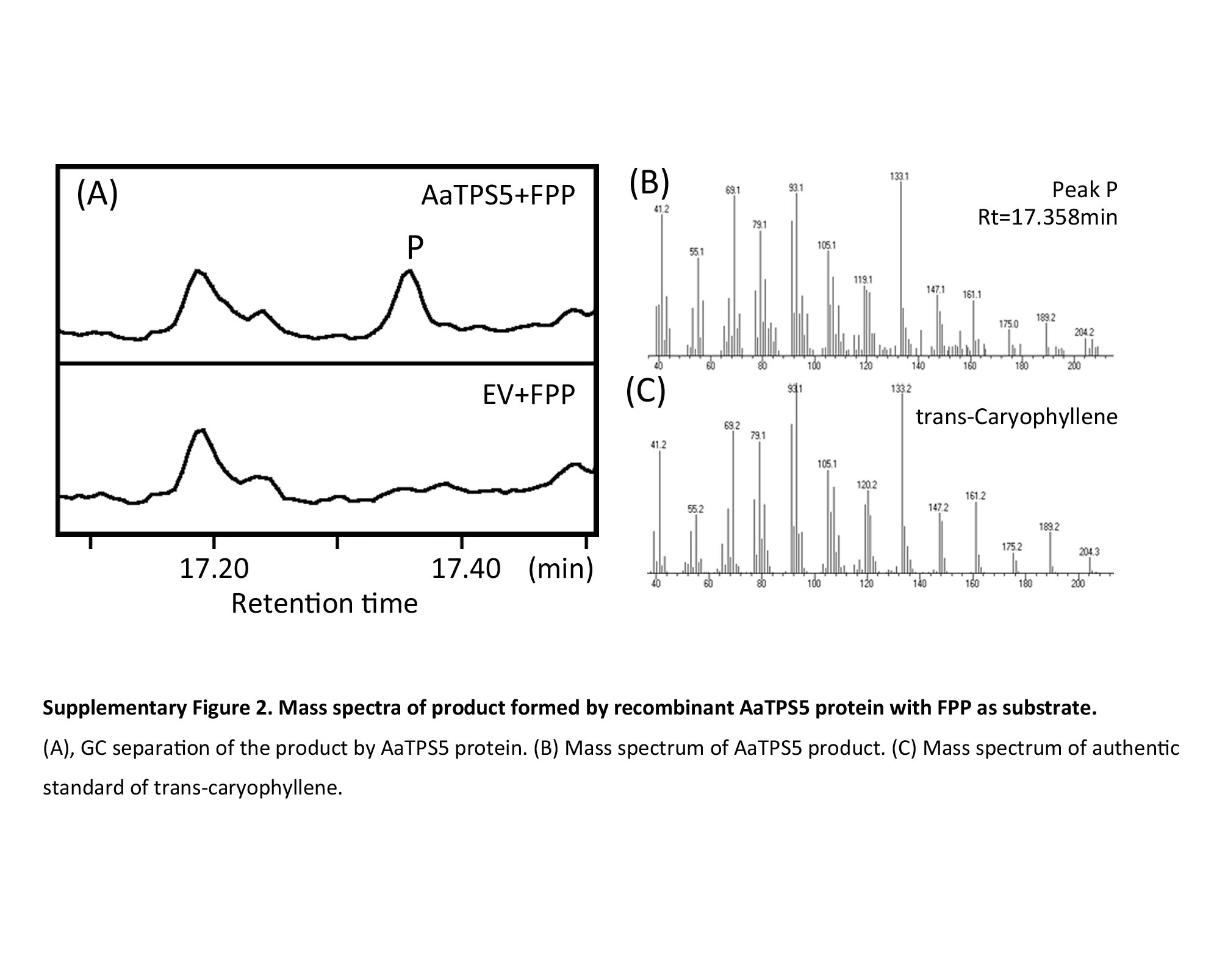

Supplement: Supplementary file 2 [file Image_2.JPEG]

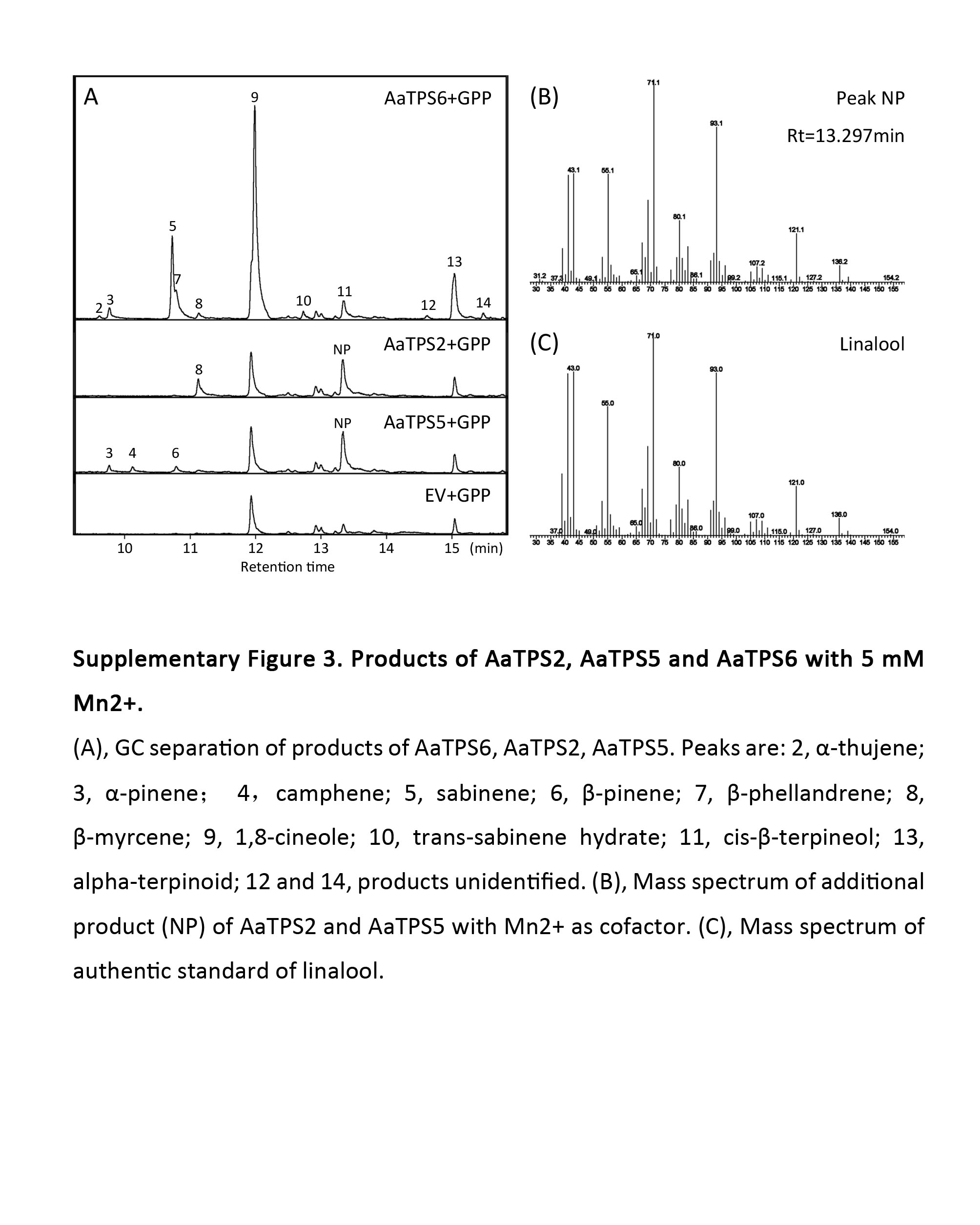

Supplement: Supplementary file 3 [file Image_3.JPEG]
