# Supplementary material for: Isolation and Characterization of Three New Monoterpene Synthases from Artemisia annua
Source: Front Plant Sci. 2016 May 10;7:638. doi: 10.3389/fpls.2016.00638 (PMC4861830; doi:10.3389/fpls.2016.00638)
Supplement: Supplementary file 4 [file Table_1.PDF]

Supplementary Table 1. Oligonucleotides used in this study.

Gene	Sequence (5'-3')	Purpose
Q_T	CCAGTGAGCAGAGTGACGAGGACTCGAGCTCAAGCTTT TTTTTTTTTTTTTT	RACE
Q_0	CCAGTGAGCAGAGTGACG	RACE
Q_1	GAGGACTCGAGCTCAAGC	RACE
AaActin-RT -S	CTTTGAACCCTAAGGCTAATC	qRT-PCR
AaActin-RT -AS	GGGCATACCCTTCGTAGATAG	qRT-PCR
AaTPS2-RT -S	TCATCCTTAATCTTCCGACTCAC	qRT-PCR
AaTPS2-RT -AS	TGCACATCTCCTTCACCGTAC	qRT-PCR
AaTPS5-RT -S	CAAGGCATACCTAGTAGAAGCAA	qRT-PCR
AaTPS5-RT -AS	GTTCCCAAATCATCAGCAAGT	qRT-PCR
AaTPS6-RT -S	CCTAAAGAAAGGGTGGGTG	qRT-PCR
AaTPS6-RT -AS	GTCATCAGCAAGTCGCAAA	qRT-PCR
AaQH1PR2	GACAAGAATACTAAGATCTC	qRT-PCR
AaQH5PR1	ATGTGAACCCGTGGAACTATC	qRT-PCR
AaQHP2	TTGGACAATGCTTGTGTATCG	qRT-PCR
AaADS-RT -S	GATCTCATGACCCACAAGGCC	qRT-PCR
AaADS-RT -AS	TTGAACTTCAAGAAACTGGCA	qRT-PCR

pET-TPS2-S	TTTCCATGGCTATGGTTAGACGAACAGCTAATTATGAGC	protein expression
pET-TPS2-AS	TTTGTCGACGTATCTCTTGAATTGGATTAAACAAC	protein expression
pET-TPS5-S	TTTCCATGGCTATG ATTAGAAGATCAGCAGGTTATCCGC	protein expression
pET-TPS5-AS	TTTGTCGACGCACCCCTTGGATTGGATTGACA	protein expression
pET-TPS6-S	TTTCCATGGCTATGGGTAGACGTTCAGCAAACTATGAGC	protein expression
pET-TPS6-AS	TTTGTCGACG CATCCCTTGGATGGGATTAATCA	protein expression

Supplementary Table 2. Terpene contents in different tissues of *A. annua* (µg/g FW).

	Inflorescence	Young leaf	Mature leaf	Stem	Root
Monoterpene	3350.3±205.1	2084.3±221.6	1287.4±61.3	78.6±24.5	2.5±0.4
Sesquiterpene	816.5±255.4	843.8±90.1	635.1±40.2	159.8±34.9	116.1±19.2
Total	4166.8±460.6	2928.2±311.8	1922.5±101.5	256.7±36.1	118.6±19.5

Organ tissues were grinded with liquid nitrogen, extracted with pentane and subjected to GC-MS for quantitative analysis with nonyl acetate as internal standard. The compounds that could not be unambiguously identified were involved in this quantification of monoterpenes and sesquiterpenes.

Supplementary Table 3. Quantification of AaTPS2, AaTPS5 and AaTPS6 product monoterpenes in *A. annua* tissues (μg/g FW)

	Inflorescence [a]	Young leaf	Mature leaf	Stem
tricyclene	16.0 [b]	12.6	8.0	0.4
α-thujene	9.0	2.0	1.0	0.0
α-pinene	48.3	70.1	33.4	2.0
camphene	265.5	212.3	142.2	5.5
sabinene	65.6	7.9	2.2	0.6
β-pinene	36.8	32.0	23.4	1.5
β-myrcene	681.6	3.8	2.1	1.1
1,8-cineole	313.1	22.2	18.1	1.9

[a] The monoterpenoids in root are too low to be identified and quantification.

[b] The content of monoterpenoids in these tissues are the mean value of three repeats.
