# Supplementary material for: Isolation and Characterization of Three New Monoterpene Synthases from Artemisia annua
Source: Front Plant Sci. 2016 May 10;7:638. doi: 10.3389/fpls.2016.00638 (PMC4861830; doi:10.3389/fpls.2016.00638)
Supplement: Supplementary file 5 [file Table_2.DOCX]

**Supplementary Table 2. Terpene contents in different tissues of *A. annua* (μg/g FW)..**

|  | Inflorescence | Young leaf | Mature leaf | Stem | Root |
| --- | --- | --- | --- | --- | --- |
| Monoterpene | 3350.3±205.1 | 2084.3±221.6 | 1287.4±61.3 | 78.6±24.5 | 2.5±0.4 |
| Sesquiterpene | 816.5±255.4 | 843.8±90.1 | 635.1±40.2 | 159.8±34.9 | 116.1±19.2 |
| Total | 4166.8±460.6 | 2928.2±311.8 | 1922.5±101.5 | 256.7±36.1 | 118.6±19.5 |

Organ tissues were grinded with liquid nitrogen, extracted with pentane and subjected to GC-MS for quantitative analysis with nonyl acetate as internal standard. The compounds that could not be unambiguously identified were involved in this quantification of monoterpenes and sesquiterpenes.
