# Supplementary material for: Isolation and Characterization of Three New Monoterpene Synthases from Artemisia annua
Source: Front Plant Sci. 2016 May 10;7:638. doi: 10.3389/fpls.2016.00638 (PMC4861830; doi:10.3389/fpls.2016.00638)
Supplement: Supplementary file 6 [file Table_3.DOCX]

**Supplementary Table 3. Quantification of AaTPS2, AaTPS5 and AaTPS6 product monoterpenes in *A. annua* tissues (μg/g FW)**

|  | Inflorescence *^a^* | Young leaf | Mature leaf | Stem |
| --- | --- | --- | --- | --- |
| tricyclene | 16.0 *^b^* | 12.6 | 8.0 | 0.4 |
| *α*-thujene | 9.0 | 2.0 | 1.0 | 0.0 |
| *α*-pinene | 48.3 | 70.1 | 33.4 | 2.0 |
| camphene | 265.5 | 212.3 | 142.2 | 5.5 |
| sabinene | 65.6 | 7.9 | 2.2 | 0.6 |
| *β*-pinene | 36.8 | 32.0 | 23.4 | 1.5 |
| *β*-myrcene | 681.6 | 3.8 | 2.1 | 1.1 |
| 1,8-cineole | 313.1 | 22.2 | 18.1 | 1.9 |

*^a^* The monoterpenoids in root are too low to be identified and quantification.

*^b^* The content of monoterpenoids in these tissues are the mean value of three repeats.
